# Report of the Committee on Surgery

**Published:** 1864-10

**Authors:** E. Andrews

**Affiliations:** Professor of Surgery in Chicago Medical College


ARTICLE XXXVI.

### REPORT OF THE COMMITTEE ON SURGERY.

By E. ANDREWS, M.D., Professor of Surgery in Chicago Medical College.

Presented to the Illinois State Medical Society. May, 1864.

### MILITARY SURGERY.

A considerable body of statistics has been accumulated by us since our last report, and we have condensed them for this report into tabular form.

The following list of cases, treated within the bounds of this State, was derived from the records of Mound City Hospital, under the care of that superior officer, Dr. H. Wardner, Surgeon U. S. Vols. The labor of arranging and preparing this list was performed by Dr. M. Block, a most valuable and enterprising Assistant-Surgeon, of the same Hospital:—

| Name of Patient. | Date of Injury. | Nature of Injuries. | Adm. to Hospt'l. | General Treatm't. | Disch'd. | Ret'd to Duty. | Died. | Wounding Instrum't. | Direction of Wounds, &c. | Remarks. |
|---|---|---|---|---|---|---|---|---|---|---|
| Case No. 1. Serg. G. F. Wallin, Co. I, 7th Iowa. | Nov. 7, 1861. | Fracture of the humerus. | " | Splint of binder's board bandages and cold water dressings. | | Jan'y. 22, '62. | | Minnie ball. | Ball passed through the inner margin of the biceps, anterior to brachial artery, fracturing the humerus and emerging at the insertion of the deltoid. | One large spicula and several small pieces of bone discharged, after suppuration had commenced. |
| Case No. 2. W. M. Kennedy, private Co. A, 7th Iowa. | " | Wounds of bones of the tarsus. | | Foot supported by splints and and bandages, dressings of cold water and arnica. | Feb'y. 20, '62. | | | Musket ball. | Struck the outer malleolus, passed between the os calcis and astragalus, emerging at the inner malleolus. | Great suppuration; disch'd almost well, but walking on crutches, on account of weakness of the injured foot. |

| Name of Patient. | Date of Injury. | Nature of Injuries. | Adm. to Hospt'l. | General Treatm't. | Disch'd. | Ret'd to Duty. | Died. | Wounding Instrum't. | Direction of Wounds, &c. | Remarks. |
|---|---|---|---|---|---|---|---|---|---|---|
| CASE No. 3. John Walgamuth, private Co. D, 7th Iowa. | Nov. 7, 1861. | Fracture of the ulna & humerus, (left side) | Nov'r. 13, '61. | Splints; bandages and cold water dressings, tonics and stimulants internally. | Feby'. 20, '62. | | | Musket ball. | Struck the fore-arm about 2 inch. below elbow-joint, fracturing the ulna, passed through the joint, splitting and fracturing lower end of the humerus, and emerged about 4 in. above joint, front side of arm. | Suppuration extensive; sufferings very great; cure perfect, save anchylosis of the joint. |
| CASE No. 4. Leander Richie, Co. B, 7th Iowa. | " | Injury of scapulæ. [coracoid process & subscapular fossa.] | " | Cold water and arnica dressings; perfect rest. | | Jan'y. 22, '62. | | | Struck about the middle of the coracoid process, passed under the subscapularis muscle, denuding the bone at this point of its periosteum, emerging below and near the inferior angle of the scapula. | Inflammation and suppuration extensive; cure perfect. |
| CASE No. 5. Jas. R. Howard, private Co. B, 7th Iowa. | Nov. 7, 1861. | Fracture of the humerus; amputation of arm; two sec'dary hemorrhages. | Nov'r. 13, '61. | Simple dressing at first; incis'n in two aneurismal culs de sac, on 3d Dec., one on brachial, another on axillary artery, followed by stimulating injections. | Mar. 6. 1862. | | | Musket ball. | Unknown; the patient being left on the field, was captured by the rebels, & while in their hands the limb was amputated. | Discharge of 2oz. of sanguinous matter on 24th Nov'r.; considerable hemorrhage on 2d of Dec.; incisions made on the 3d, (see general treatment.) Tumor formed on dorsum of scapula, Jan. 30th, discharging matter for 2 weeks; cure perfect. |
| CASE No. 6. Patrick Welsh, private Co. K, 22d Ill. Inf'ty. | " | Wound of the thigh with dinudation of perios- | Nov'r. 12, '61. | Bandages, cold water dress'gs, tonics, stimulants, and generous diet. | | | Jan. 1, 1862. | Minnie ball. | Ball entered the outer side of left thigh, near junction of upper and middle third; another entered same leg 2 inch. below knee-joint; | Suppuration involving muscles of thigh and leg; extremely offensive; on *post mortem* examina'n, entire dis- |

| | | | | | | | | | | |
|---|---|---|---|---|---|---|---|---|---|---|
| | | teum of femur. | | | | | | | neither ball could be found with the probe. | organization of soft parts and dinudation of periosteum of femur was discovered. |
| CASE No. 7. J. M. Hammitt, Serg. Co. F, 7th Iowa. | " | Fracture of femur (middle third.) | Nov'r. 13, '61. | Cold water and arnica dressing; extension and box spl'ts. | March 22, '62. | | | Minnie ball. | Ball entered the outer side of left thigh, passed thro', emerging on inner side, a little lower than the point of entrance; fracturing in its passage the femur at the middle third. | A first emollent to 3 times its natural size; 15 days after, secondary hemorrhage from a branch of profunda artery, with considerable loss of blood, arrested by tourniquet, and ice water; suppuration extensive for 3 months; several spiculæ of bone discharged; cured; shortening half an inch; bone united March 11th. |
| CASE No. 8. Jas. M. B. Gaston, private Co. C, 22d Ill. Infantry. | " | Flesh wound of the thigh with slight injury to the femur | " | Cold water dressing. | | Furloughed Jan. 22, '62. | | Minnie ball. | Entered left thigh, about 1 inch anterior and above the external condyle of femur, emerging through tendon of rectus femoris muscle. | Extensive suppuration leading to fear of serious injury to bone. |
| CASE No. 9. David Clammer, Serg. Co. C, 2d Iowa Infantry. | " | Fracture of femur, upper 3d. | " | Extension splint and simple dressing. | | Jan. 22, '62. | | Musket ball. | Ball entered about 3 inches below the trochanter major through sartorius muscle, fracturing the bone and emerging on the opposite side. (?) | Suppuration very copious. |
| CASE No. 10. John R. Kell, Serg. Co. G, 22d Ill. Infantry. | " | Fracture of the occipital bone. trephin'd | | Depressed bones removed by trephine, serrated edges trimmed, and flaps dr'wn together by silver sutures. | | Furloughed Jan. 22, '62. | | Ball (?) | Ball struck and broke the occip. bone, about 1 inch to the right of lateral sinus. | Both plates of the bone were depressed and a spicula of the inner plate driven into the substance of the brain. The patient has since been discharged on account of epilepsy, to which he had not been subject before. |

| Name of Patient. | Date of Injury. | Nature of Injuries. | Adm. to Hospt'l. | General Treatm't. | Disch'd. | Ret'd to Duty. | Died. | Wounding Instrum't. | Direction of Wounds, &c. | Remarks. |
|---|---|---|---|---|---|---|---|---|---|---|
| CASE No. 11. Chas. Koch, private Co. G, 22d Ill. Infantry. | Nov. 7, 1861. | Flesh wound of thigh, injuring the femur (middle third.) | Nov. 13, '61. | Cold water dressing and poultices. | Aug't. 27, '62. | | | Minnie ball. | Ball entered right thigh on outer side and middle 3d, passed under the femur, denuding it of periosteum, and was lost in the muscular substance. | Suppuration very copious but never disagreeable; when rec'd into Hospital the patient's clothes had not been changed or his wound dressed; leg somewhat painful, causing patient to walk lame when discharged. |
| CASE No. 12. John Morgan, Co. (?) | " | Flesh wound of thigh, lower 3d. | " | Cold water dressing, milk-punch—generous diet, &c. | | | | Musket ball. | Ball entered thigh, on the outer side, 4 in. above the patella, passing between vasti and rectus muscles, emerging from the opposite side, 1 inch above point of entrance. | Suppuration very great; cure perfect. |
| CASE No. 13. E. M. McCarty, ord. serg. Co. C, 22d. Ill. Inf'ty. | " | Flesh wounds of both thighs, upper 3d. | " | Cold water and arnica dressing. | | Furlo'. Jan. 22, '62. | | Musket ball. | Ball entered the outer side of right thigh, 4 in. below perineum, passing through both thighs, behind femur. | Suppuration very extensive; both wounds nearly healed, Jan. 22, '62; returned to reg't. as 2d lieutenant, but was obliged to resign, on acc't of weakness of both limbs. |
| CASE No. 14. Wm. Miller, (?) | | Flesh wound in the back. | " | " | | | | Rifleball. | Ball entered a little external to the middle portion of the axillary border of scapula, passing through the teres major, downwards & across, through the trapezius, and was extracted near the 1st lumber vertebra. | Great suppuration; recovery perfect. |
| CASE No. 15. Charles Wilbert, priv. Co. B, 7th Iowa Infantry. | " | Flesh wound of the thigh. upper 3d. | " | " | | Jan'y. 22, '62. | | Minnie ball. | Ball entered the thigh opposite the trochanter major, passed down the side of femur, and was lost in the muscular substance. | Suppuration extensive. |
| CASE No. 16. Mathias O'Blennis, Co. E, 7th Iowa Infantry. | " | Flesh wounds of nates, making 4 holes. | " | " | Feb'y. 15, '62. | | | Ball. | Ball passed through the nates, from right to left. | Speedy convalescence; cure perfect. |

| | | | | | | | | | | |
|---|---|---|---|---|---|---|---|---|---|---|
| Case No. 17. L. Van Hoosen, priv. Co. D, 7th Iowa Infantry. | " | Flesh wounds of both thighs; upper 3d | " | Cold water and arnica dressing. | | Jan'ry 1, '62. | | | Ball passed in a direct line through both thighs at the lower end of upper third, posterior to the femur. | Cure perfect, save a slight contraction of the flexor muscles of both legs. |
| Case No. 18. Wm. Eshmell. | " | Flesh wound of foot. | " | " | | | | | Ball entered near and under the middle of the 1st metatarsal bone of right foot, passing under the deep fascia, and made its exit through lower end of tendo achillis. | Cure perfect. |
| Case No. 19. Alb't Hite, priv. Co. C, 7th Iowa Infantry. | " | Wound of right lung. | " | Compresses, bandages and cold water dressing, tonics and stimulants. | | | Nov'r. 24, '61. | Ball. (?) | Ball entered 3 inches below right scapula, grazing the spine, passing through the body, emerging about 3 in. below and to the right of right nipple. | Suppuration very great, pus dark, watery and offensive; air passed through both orifices of wounds when undressed. |
| Case No. 20. D. Wallis, priv. Co. E, 7th Iowa Infantry. | " | Wound of left lung. | " | Aconite, pulv. Dov. and camphur; generous diet and simple dressings. | Feb'ry 20, '62. | | | Ball. (?) | Ball entered 1 inch from outer margin of left scapula, passed in thoracic cavity, wounding left lung. | Expectoration of blood during 4 days; completely arrested on the 6th; cure in 7 weeks. |
| Case No. 21. Wm. L. Wood, priv. Co. H, 7th Iowa Infantry. | " | Wound of right lung. | " | | | | Dec'r. 1, '61. | Minnie ball. | Ball passed through the right scapula into the thoracic cavity, through upper lobe of right lung; emerged between 4th and 5th ribs, fracturing the 4th. | Pulse 120; breathing short and quick; expectorations bloody. |

| Name of Patient. | Date of Injury. | Nature of Injuries. | Adm. to Hospt'l. | General Treatm't. | Disch'd. | Ret'd to Duty. | Died. | Wounding Instrum't. | Direction of Wounds, &c. | Remarks. |
|---|---|---|---|---|---|---|---|---|---|---|
| CASE No. 22. Chas. Hoffman, priv. Co. K, 7th Ill. Inft'y. (17 years of age.) | At Fort Don'n. Feb'y. 14, '62. | Fracture of tibia and fibula. | | Primary & secondary amputation of leg. | March 15, '62. | | | Musket ball. | Unkown; amputation having been performed on the battle field. | After the 2d operation the appearance of the limb improve daily; recovery perfect. |
| CASE No. 23. Wm. Casey, Co. K, 1st Mo. Art. | " | Fracture of fibula with destruction of calf of leg. | | Bandages, tonics, stimulants. &c. | | | Feb'y. 28, '62. | Shell. | Shell carried away the greatest part of the calf, and fracturing the fibula by concussion. | Constitutional irritation extreme; patient died of traumatic tetanus. |
| CASE No. 24. Geo. Smith, seaman, Gunboat Pittsburg. | " | Wound of back. | Feb'y. 21, '62. | Emolient poultice on wounded parts; tonics, stimulents, &c. | | | March 20, '62. | Piece of iron, st'v-pipe. | Two wounds near posterior margin of axilla extending upwards one inch beneath the skin for about 2 inches. | 12 hours after his admission to the hospital the missile was removed; sero-purulent discharges, on admission, and the parts highly tumefied and painful. |
| CASE No. 25. W. Cool Russel, Co. E, 9th Ill. Inft'y, (aged 17 years.) | " | Fracture of occipital bone. | | Trephined, and detached and depressed portions of bone removed. | March | | | Minnie ball. | Ball struck the occipital bone near its junction with the left parietal, depressing doth plates. | When rec'd in hospital, his pulse was 65; 24 hours after operation breathing was natu'al, recov'y rapid & good. |
| CASE No. 26. Dennis Bryan, Co. I, 18th Ill. Infantry. | " | Fracture of inferior maxilla'y bone | " | Resect'n of bone from symphysis to the groove for fascial artery; sutures, cold water dressing, &c. | April 28, '62. | | | " | Ball struck the chin, glancing and following the bone to within one inch of the angle, when it passed out, crushing the entire bone on its way. | Wound healed rapidly; and bones united by cartilage, cure perfect. |

| | | | | | | | | | | |
|---|---|---|---|---|---|---|---|---|---|---|
| CASE No. 27. Charles Mervin, seaman, gunboat Pittsburg. | " | Fracture of frontal bone, depression of both plates. | Feb'y. 20, '62. | The surgeon of the boat, mistaking it for a flesh wound, treated it as such, with adhesive strips, &c. | | | Feb. 24 1862. | Shell. | Fragment of shell struck the frontal bone, about 2 inch. above right eye. | Patient wal'd from boat to hosp. on the 20th; felt well until the 24th when he commenced vomiting, became delirious; had a stertous breathing, dialated pupils, &c.; died 2 hours after attack. *Post mortem* examination: fract're of frontal bone depression of both plate, spicula of bone in the substance brain, both outside the dura-mater; brain disorganized and softened. |
| CASE No. 28. Wm. Randolphe, Co. C, 25th Ind. Infantry. | Feb'y. 15, '62. | Fracture of femur, middle3d | " | Amputation primary & secondary, flap operation. | | | Feb. 24 1862. | | Unknown; the patient's limbs having been amputated on the field. | After coming in hosp. flaps retracted, sutures sloughed, expos'g 1 in. of the bone, at the end of which an osseous formation was found; the exposed portion of bone was resected and the flaps drawn togeh'r |
| CASE No. 29. J. Strickland, Co. D, 8th Ark. (rebel.) | " | Fracture of superior maxil'ry bone | | Extraction of ball in two hal's at an interval of 4 w'ks. The 1st was extrac'd by incision fr'm the posterior margin of the sterno - cleido muscle, and the 2d from the face to the point of entrance of the ball. | March 6, 1862 sent to Camp Douglas, Chicago. | | | Musket ball. | Ball entered the face about 1 inch below the right eye, fracturing supr. maxillary bone and driving a spicula of bone in the antrum of highmore, then deflecting it passed under the skin and cellular tissue over the angle of the jaw through the sterno cleide muscle, 3 inches below the mastoid process. | Excessive inflammatory action 4 weeks after 1st extraction, when the second portion of the ball was removed; after this the wound healed rapidly. |

| Name of Patient. | Dute of Injury. | Nature of Injuries. | Adm. to Hospt'l. | General Treatm't. | Disch'd. | Ret'd to Duty. | Died. | Wounding Instrum't. | Direction of Wounds, &c. | Remarks. |
|---|---|---|---|---|---|---|---|---|---|---|
| CASE No. 30. Michael Kelley, seaman, Gunboat Louisville | Feb'y. 16, '62. | Flesh wound of shoulder & back. | Feb'y. 21, '62. | Adhesive strips, cold calendula dressing, tonics, stimulants and rich diet. | | July 4, 1862. | | Fragm't. of shell. | Wound extending from the middle of deltoid muscle, backwards & downwards to a point opposite the 2d dorsal vertebra. | Wound about 10 inches in length, gaping from 1 to 4 inches, laying bare left scapula and ribs, along the track. When wounded he was unaware of having received any serious injury; had to be ordered to retire. Inflammation, œdema and suppuration extensive.—Cure perfect. |
| CASE No. 31. Julius Schusker, 48th Ill. Inft'y. | " | Comminuted fr. of radius and ulna, tra'matic tetanus. | " | Spicula of bones removed and limb dressed.—Chloroformed to overcome the rigidity of muscles, more or less for 48 hours | March 7, '62. | | | Minnie ball. | | When admit'd wounds looked well, the bones having been properly adjusted, & the patient's appetite was good. On the 24th, tetanus supervened, being ushered in by fever, closure of jaws and partial immobility of limbs and trunk, with difficult deglutition. Doing well 2 weeks after the attack when he was furlo'ed. |
| CASE No. 32. Geo. W. Morgan, seaman, Gunboat Carondolet. | " | Flesh wound of back and tra'matic tetanus. | " | Wound dressed as usual. Chloroformed at intervals from ½ to 1 hour during 4 days. | | | March 9, '62. | Fragm't. of shell. | Entered at the point of the scapula, passing upwards between this bone and the cellular tissue, making a wound three inches in length. | Doing well for 8 days, when a small piece of cloth was extracted from the wound; 4 days thereafter tetanus supervened, which continued for four days, when the patient died from exhaustion. |

| | | | | | | | | | | |
|---|---|---|---|---|---|---|---|---|---|---|
| Case No. 33. Stephen Burrows, 20th Ill. Infantry. | Battle of Ft. Donelson. Feb'y. 15, '62. | Fracture of femur (?) followed by traum'tic tetanus. | " | Dressings as usual. *Blister over the whole length of spine. Strychnia, opium, & camphor. | | | Mar. 2, 1862. | (?) | (?) | Doing well until Feb. 30, when all the symptoms of tetanus supervenes, and the patient died 28 hours after the attack. The remedies employed could not arrest the progress of the disease. |
| Case No. 34. R. N. Fowler, Co. A, 31st Ill. Infantry. | " | Fracture of tibia, traum'tic tetanus. | " | Dressings as usual. *Chloroformed more or less for 36 h'rs. | Sent home on a furlo'. | | | Minnie ball. | Enter'd the leg, fracturing slightly the tibia, and glanced out, doing very little injury to the soft parts. | Doing well until Feb. 29th, when symptoms of tetanus came on gradually, increase in violence. Cure perfect. |
| Case No. 35. G. Arnold, capt. Co. H, 24th Ohio Vols. | Battle Pittsburg Landing. Apr. 7, 1862. | Woundof scapula andneck sec'dary hemorrhage. | (?) | Simple dressing and generous diet. | | (?) | | Minnie ball. | Ball entered left scapula at a point opposite the third dorsal vertebra, passed obliquely upwards, lodging in inner margin of the right sterno-cleido muscle, about 1 in. below pomum adami. | Supporation great at point of entrance, white wound at the point of exit, healed by first intention. Expectoration a little bloody at first and difficulty of deglutition. Doing well April 20th. Cure perfect. |
| Case No. 36. Colby Shrader, Co. I, 17th Kentucky Infant'y. | " | Woundof shoulder andneck | | Simple dressings; tonics, stimulants, &c. Unsuccessful attempt by Prof. Gross to tie artery, subclavian. | | | April 18, '62. | Minnie ball. | Ball entered deltoid muscle, about 3 in. above its insertion into humerus, passed upwards, striking inner head of that bone, then glancing inwards & backwards bet. pectoralis major and minor muscles; passed between clavicle and first rib, over track of subclavian vein, making its exit on opposite side, between scalenus anticus and rectus capitis anticus major, wounding probably the subclavian artery in its passage. | Ten days after the wound was received secondary hemorrhage occurred sufficient to lead to the belief that it proceeded from the subclavian artery. An unsuccessful attempt at tying the artery was made by Professor Gross, who was at that time visiting the Hospital. A sack filled with a coagulum fully the size of a hen's egg was found resting on the pluera. |

* These treatme'ts of course, were commencedwhen the symptoms of tetanus were developed.

| Name of Patient. | Date of Injury. | Nature of Injuries. | Adm. to Hospt'l. | General Treatm't. | Disch'd. | Ret'd to Duty. | Died. | Wounding Instrum't. | Direction of Wounds, &c. | Remarks. |
|---|---|---|---|---|---|---|---|---|---|---|
| CASE No. 37. William Merlin, private Co. A, 41st Ill. Inft'y. | April 7,1862. | Woundof of right lung. | | Simple dressings; aconite, camphor, and Dover's powd'r, with perf't rest. | May 7, 1862. | | | Minnie ball | Ball entered chest bet. 2d and 3d rib, about 1 in. to right of sternum, passed obliquely through upper lobe of right lung, then thro' pectoralis major & minor muscles, striking & fissuring the humerus; it made its exit about 1 in. above insertion of deltoid muscle. | Bloody expectoration for the first four days. Result—entire convalescence. |
| CASE No. 38. T. H. Simmons, leut. Co. F, 14th Ill. Inft'y. | " | Woundof the right lung and scapula. | (?) | Simple dressings; perfect rest. | May 8, 1862. | | | Minnie ball. | Ball passed between 2d and 3d ribs, 2 inch. to the right of sternum, through the upper lobe of right lung and thro' the scapula. | Bloody expectoration for first 8 days after receiving injury; constitutional irritation slight; pulse 100. 11th day, pulse 80, appetite good, and symptoms favorable. May 8th, wounds closed by granulation; no pain in the chest; draws full inspirations. |
| CASE No. 39. A. A. Veach, private Co. I, 28th Ill. Inft'y. | April 6, '62. | Woundof left lung | (?) | " | | | April 19, '62. | Ball (?) | Ball entered the fourth intercostal space, 4 in. left to sternum, and emerged between 8th and 9th ribs, about 3 inches to the right of the vertebral column. | Patient, when wounded, was inclined forwards. Lung highly inflamed; respiration difficult; expectoration bloody. Bloody serum discharge from wound for 3 days, after which an exceedingly offensive, very profuse, dark-colored, thin, excoriating, purulent discharge took place. Appetite remained good throughout, and bowels were inclined to be constipated. |

| | | | | | | | | | | |
|---|---|---|---|---|---|---|---|---|---|---|
| CASE No. 40. G. W. Crabtree, private Co. C, 11th Ill. Inft'y. | Apr. 7, 1862. | Wound of descending col'n. | (?) | Upright position, cold water dressings and saline cathartics. | | June 27, '62. | | | Ball entered the abdomen, 3 inch. to the left of the umbilicus, passed through the body, emerging about 1 inch. to the left of the spine. | Fecal discharge from both wounds until May 2d, when the wounds healed and the bowels acted regularly; when the bowels were constipated the discharge from the wounds was profuse & attended with much pain; when the bowels were relaxed discharge was slight and the patient comfortable; cure perfect. |
| CASE No. 41. G. W. Spalding, private Co. D, 52d Ill. Inft'y. | " | Fracture of right clavicle. | | Exsection of the sternal half; arnica water dressing, stimulant, generous diet, and anodynes at bedtime. | | | May 7, 1862. | Minnie ball. | Ball struck the centre of right clavicle, shattering the bone, and became embedded under the clavicle near its sternal end. | April 19th, several spiculæ of bone, about $\frac{1}{3}$ of an oz. ball, and several small pieces of clothing were removed from the wound. 22d, general condit'n good, pulse 90; exsection performed by Surgeon Franklin; operation lasted 20 minutes, with a loss of less than 1 oz. of blood; a brass button belonging to the coat of the patient was found embedded under the bone, near the acromion process, and removed. |
| CASE No. 42. Adam Sheldon, private Co. A, 43d Ill. Inft'y. | " | Wound of knee-j't. | | Cold water dressings and bandages after inflammation was reduced; tonics, stimulants, and generous diet. | July, | | | | Ball passed through the patella, into the joint, where it could not be found. | Suppuration excessive; abcesses formed around the knee-joint; inflammation and swelling extending up to the thigh and down to the foot; cured with anchylosis of the knee-joint. |

| Name of Patient. | Date of Injury. | Nature of Injuries. | Adm. to Hosptl. | General Treatm't. | Disch'd. | Ret'd to Duty. | Died. | Wounding Instrum't. | Direction of Wounds, &c. | Remarks. |
|---|---|---|---|---|---|---|---|---|---|---|
| CASE No. 43. Edw. Hawkins, private Co. F, 52d Ill. Inft'y. | Apr. 7, 1862. | Fracture of femur left, low. third. | | Limb adjusted, spicula of bone removed, splint, extension, and cold water dressings. | July, 1863. | | | | Ball entered on outer side and lower third of left thigh, making a cominuted fracture of the femur, emerged on inner side, and passed through left thigh posterior to the femur. | Right leg healed rapidly; bone united on 27th June, (50 days after injury); there seemed however to be some necrosed bone, which kept up continued irritation; remained in hospital over a year, when he was disch'd the service at his request. |
| CASE No. 44. Saml. M'Combs, private Co. D, 23d Ill. Inft'y. | " | Fracture of tibia and fibula, near the tubercle. | | Amputation of the thigh at the lower third. | June, (?) | | | | | Six days after injury was received, the wound had become gangrenous, necessitating an amputation. May 15th, symptoms of pyemia made their appearance, but the disease was checked by tonics and stimulants, under the influence of which he slowly recovered; cure perfect. |
| CASE No. 45. John W. Milton, private Co. E, 14th Iowa Infy. | " | Fracture of knee-joint. | | Limb adjusted, splint extension by cord and pulley, cold water dressing, tonics, stimulants, and generous diet. | | | Sept. 15, '62. | | | May 1st, patient doing well; 3d, opened an abscess 1 ½ inch. below patella, discharging considerable quantity of pus; 27th, removed two small pieces of lead from lower part of the patella; bone partially united; June 7th, large suppuration of knee-joint discharging a quanity of pus and a small piece of the lining of the pants; 11th, complete union, all symptoms favorable; 28th, able to walk about; July 2d, diarrhœa followed by symptoms of pyemia, from which date the patient gradually sunk daily until he died. |

### RESUME.

| Nature of Injuries. | Number Total. | Battle of Belmont. | Battle of Pittsburg Landing. | Battle of Fort Donelson. | With Amputation. | Result. Returned to Duty. | Discharged. | Died. | Without Amputation. | Result. Returned to Duty. | Discharged. | Died. |
|---|---|---|---|---|---|---|---|---|---|---|---|---|
| Fracture of humerus, | 2 | 2 | 0 | 0 | 1 | — | 1 | — | 1 | 1 | — | — |
| " " femur, | | | | | | | | | | | | |
| " " upper third, | 1 | 1 | 0 | 0 | 0 | 0 | 0 | | 1 | 1 | — | — |
| " " middle " | 2 | 1 | 0 | 1 | 1 | 0 | 0 | 1 | 1 | — | 1 | — |
| " " lower " | 2 | 0 | 2 | 0 | 0 | 0 | 0 | 0 | 2 | 0 | 1 | 1 |
| Not known, | 1 | 0 | 0 | 1 | 1 | 0 | 0 | 1 | 0 | 0 | 0 | 0 |
| Fracture of ulna and humerus, | 1 | 1 | 0 | 0 | 0 | 0 | 0 | 0 | 1 | 0 | 1 | 0 |
| " " ulna and radius, | 1 | 0 | 0 | 1 | 0 | 0 | 0 | 0 | 1 | 0 | 1 | 0 |
| " " tibia, | 1 | 0 | 0 | 1 | 1 | 0 | 1 | 0 | 0 | 0 | 0 | 0 |
| " " fibula, | 1 | 0 | 0 | 1 | 0 | 0 | 0 | 0 | 1 | 0 | 0 | 1 |
| " " tibia and fibula, | 2 | 0 | 1 | 1 | 2 | 0 | 2 | 0 | 0 | 0 | 0 | 0 |
| " " sup'r. maxillary, | 1 | 0 | 0 | 1 | 0 | 0 | 0 | 0 | 1 | 0 | 1 | 0 |
| " " inf'r. " | 1 | 0 | 0 | 1 | | 0 | 0 | | | | 1 | |
| " " occipital bone, | 2 | 1 | 0 | 1 | | 0 | 0 | 0 | | 1 | 1 | 0 |
| " " frontal " | 1 | 0 | 0 | 1 | | | | 0 | | | | 1 |
| Injury to bones of tarsus, | 1 | 1 | 0 | 0 | | | | | | | 1 | |
| " " periosteum femur, | 3 | 3 | 0 | 0 | — | — | — | — | 3 | 1 | 1 | 1 |
| " " " scapula, | 1 | 1 | 0 | 0 | | | | | 1 | 1 | | |
| Fracture of clavicle, | 1 | 0 | 1 | 0 | 1 | | | 1 | | | | |
| Wound of knee-joint, | 1 | 0 | 1 | 0 | — | | | | 1 | | 1 | |
| " " right lung, | 4 | 2 | 2 | 0 | | | | | | | 2 | 2 |
| " " left " | 2 | 1 | 1 | 0 | | | | | | | 1 | 1 |
| " " desc'g. colon, | 1 | 0 | 1 | 0 | | | | | | 1 | — | — |
| " " shoulder and neck, | 3 | 0 | 2 | 1 | | | | | | 2 | — | 1 |
| " " back, | 3 | 1 | 0 | 2 | | | | | | 1 | 0 | 2 |
| " " nates, | 1 | 1 | | | | | | | | | 1 | |
| " " foot, | 1 | 1 | | | | | | | | 1 | 0 | 0 |
| " " one thigh, | 2 | 2 | | | | | | | | 2 | | |
| " " both thighs, | 2 | 2 | | | | | | | | 2 | | |
| | 45 | 21 | 11 | 13 | 7 | 0 | 4 | 3 | 14 | 14 | 14 | 10 |

Combining these statistics with those gathered by your Committee from other western battle fields, we obtain the following tabular results:—

*Wounds Received in the Western Battles.*

| | Recov'd. | Died. | Total. |
|---|---|---|---|
| Gunshot Fractures of the Cranium, | 3 | 8 | 11 |
| " " " Face, | 15 | 2 | 17 |
| " " " Shoulder-Joint, | 4 | 2 | 6 |
| " " " Humerus, | 30 | 5 | 35 |
| " " " Elbow-Joint, | 14 | 3 | 17 |
| " " " Fore-arm, | 20 | | 20 |
| " " " Pelvis, | 2 | | 2 |
| " " " Femur, | 17 | 16 | 33 |
| " " " Knee-Joint, | 9 | 16 | 25 |
| " " " Leg, | 23 | 9 | 32 |
| Penetrating Wounds of Thorax, | 12 | 20 | 32 |
| " " " Abdomen, | 4 | 12 | 16 |

*Fractures of the Cranium.*

Three of these only recovered, and these were merely ploughed by the shot, the bullet not entering the brain. Two of them were trephined for the depression. None of the cases of penetrating wounds were trephined, as the bullet and fragments of bone, hair, and clothing are in such cases driven far within the cerebrum, and cannot be removed by operation.

*Fractures of the Face.*

There is but little mortality from this accident.

*Fractures of Knee-Joint.*

The great mortality of this injury is obvious at a glance, being about 66 per cent. Many cases were lost early in the war by the reluctance of surgeons to amputate. The innocent look of a knee-joint, which has been penetrated by a bullet, should, however, never deceive us. The patient will die unless he has operative assistance. We have been able to learn of only two cases which recovered without operation.

*Penetrating Gunshot Wounds of the Thorax.*

Extremely dangerous, only 12 out 32 recovering.

*Penetrating Gunshot Wounds of the Abdomen.*

Still more fatal, only 25 per cent. recovering.

*Amputations.*

The following cases of amputation are on our records:—

| | Recov'd. | Died. | Total. |
|---|---|---|---|
| Amputation at shoulder-Joint, | 4 | 2 | 6 |
| " of the Arm, | 14 | 1 | 15 |
| " " Forearm, | 5 | | 5 |
| " " Thigh, Upper third, | 6 | 4 | 10 |
| " " " Middle " | 4 | 7 | 11 |
| " " " Lower " | 10 | 5 | 15 |
| " " Leg, | 21 | 4 | 25 |

*Resections.*

A considerable number of cases of resections have come under our observation with the following results:—

| | Recov'd. | Died. | Total. |
|---|---|---|---|
| Resection of the Shoulder-Joint, | 8 | 2 | 10 |
| " " Knee " | 1 | | 1 |
| " Continuity of the Shaft of Femur, | | 2 | 2 |
| " of the Elbow-Joint, | 6 | 1 | 7 |

*Discussion of the Operations.*

It is now well settled that amputations of the superior extremity should only be performed when the limb is obviously going to mortify, from the destruction of its vessels and nervous trunks. Shattered shoulder and elbow-joints should be resected instead of amputated. The mortality of amputation at the shoulder, as above shown, is one in three, while that of resection at the same place is only one in four. In the British wars with Napoleon, 44 cases of amputation at the shoulder are reported, of which 17 died. In the Schleswich-Holstein campaign, 19 resections of the shoulder are reported, of which 7 died. Combining these and our own statistics, we have the following results:—

| | Recov'd. | Died. | Total. | Pr ct. of Deaths. |
|---|---|---|---|---|
| Amputation at the Shoulder, | 31 | 19 | 50 | 38 |
| Resection " " | 20 | 9 | 29 | 31 |

Showing an advantage of 7 per cent. in favor of resection.

Amputations of the arm below the shoulder have but little mortality. Of 15 cases only 1 died, and of 72 cases mentioned by GUTHRIE, only 17 died. Combining both, we have a mortality of a little over 20 per cent. The British mortality is obviously excessive, owing to the crowded state of their hospitals in the Crimea and Scutari. If the men are kept in open tents in the field, the mortality of this amputation will not be much above 8 or 10 per cent. Still the arm should not be amputated for a gunshot fracture, unless the circulation is obviously destroyed, as it recovers from the most surprising injuries with ease. In cases of badly shattered elbow-joints, resection should be preferred to amputation. The mortality in our cases of this resection was only 1 in 7. ESMARCH quotes 40 cases, of which 6 died. Combining these, and comparing the result with the amputations of the arm before mentioned, we have the following table:—

| | Recov'd. | Died. | Total. | Pr ct. of Deaths. |
|---|---|---|---|---|
| Resection of the Elbow-Joint, | 40 | 7 | 47 | 15 |
| Amputation of Arm, | 69 | 18 | 87 | 21 |

Showing an advantage of 6 per cent in favor of resection of the elbow.

Amputations of the fore-arm present very little danger, but are rarely necessary except for cases where the hand has been torn off. In nearly all bullet wounds the hand may be saved.

The statistics of this war present some anomalies in the matter of amputation of the thigh. Thus, for instance, out of 10 amputations in the upper third, only 4, that is 40 per cent., were fatal, whereas, in military experience heretofore, the mortality of amputations at that locality have been 80 to 90 per cent. In the Crimean war it was 87 per cent. This anomaly in the statistics occurs in consequence of adding in all the high amputations of the thigh in a certain division of troops at the seige of Vicksburg. The wounded in this division were not transported to general hospital, but were treated in the field in open tents, pitched on a high, breezy bluff. The curtains of the tents being raised, the men breathed a pure untainted air, and made recoveries which would astonish the denizens of crowded brick hospitals. We are informed by surgeon JOHN M. WOODWORTH, of the 1st Illinois Light Artillery, that a similar superiority of success accompanied the field treatment of the wounded near Atlanta. Our own personal experience in the field also points in the same direction. Indeed we are of the opinion that there is seldom any hospital arrangement which gives so favorable results after amputation as retaining the men in the fresh air of the fields. The amputations at the middle third of the thigh contain no Vicksburg statistics, and are made up of men mostly treated in general hospitals. The results, therefore, are less favorable, and actually show a mortality of 64 per cent. The result of our experience is, that the difference in mortality between the results of good field treatment, and those of treatment in average general hospital buildings is nearly as follows:—

| Mortality of amputation of the thigh | Treated in Ordinary General Hospital Buildings. | Treated in the Field in Good Circumstances. |
|---|---|---|
| At the Upper third, | 85 per cent. | 45 per cent. |
| " Middle " | 60 " | 30 " |
| " Lower " | 30 " | 20 " |

It is gratiying to observe, however, that many of our military hospitals have been so far improved that they now rival the success of field treatment, and that on the whole our present general hospitals are superior to any that have ever been constructed on so large a scale, in European wars. It is important also to observe that the essence of the improvement, consists in the superior character of modern arrangements for ventilation. In the Mower General Hospital, at Chestnut Hill, Philadelphia, these arrangements are particularly excellent, and the air is freely admitted to the wards, close to the head of each cot, so that every patient enjoys a respiration almost as pure as that of the open fields. The consequence is, as might be expected, out of 6000 patients no case of hospital gangrene has occurred, and only one death has taken place from erysipelas. In short, hospital gangrene, erysipelas, and pyæmia, the special scourges of hospitals, have been complely disarmed of their terrors.

CIVIL SURGERY.

This department of our science has been making important advances. The improvements in Ophthalmic Surgery will be detailed by the Committee on that subject. In syphilitic literature, we have to note the pretty general acquiescence of the profession, in the opinion that hitherto we have confounded two distinct diseases under the name of syphilis. The Hunterian Chancre instead of being a typical form of either, is the result of a complication of chancre with chancroid. The subject cannot, however, be fully discussed in this report. The best elucidation of the subject, in English, is at present found in BUMSTEAD on Venereal; last edition. We dissent, however, from some of his conclusions on the treatment of syphilis.

*Diseases of the Articulations.*

There has been within the past few years, a general advance of the art of treating joint and spinal diseases.

There are three great principles which run through this whole branch of surgery, like lines of light, which, when fully comprehended, reduce its apparent obscurity to clearness and order. These principles are as follows:—

1st. Simple chronic inflammation of the joints and spine have a tendancy to spontaneous recovery, but this tendency is overcome by the irritating influence of the pressure and friction of the parts upon each other, by which the inflammation is exasperated and forced on to the destructive results of suppuration and caries. Hence a vast proportion of the spinal, hip, and knee diseases are readily cured, by any good extending apparatus which draws sufficiently to take off all the pressure and friction of the inflamed parts.

2d. The plastic diathesis, when strongly present, has an almost unlimited power to prevent suppuration and caries; while the supervention on the other hand of the aplastic diathesis, will often bring a joint to these destructive results in a very few days. Now, as modern science has placed these diathesis very much within our control, we put off the suppurative stage, and gain unlimited time and opportunity for the cure of joints, by maintaining a decidedly plastic condition in our patients.

3d. All tissues, bone included, yield and change their form under continuous mechanical pressure or tension. Hence not only club-feet, but bow-legs, bent-knees, flexed-hips, and curved-spines, as well as almost all other deformities of position, will yield to mechanical force steadily applied, and very many cases which were formerly reckoned hopeless, are perfectly curable. On these three simple principles hang almost all the resources of modern orthopedy.

The subject of fractures has received, of late, increased attention among Western surgeons. Dr. PRINCE, of Jacksonville, Illinois, Dr. LATTA, of Goshen, Indiana, Dr. DODGE, of Janesville, Wisconsin, and others, in various locations, have developed improved appliances of value. Perhaps the most practically useful of them are those which have been devised to secure painless counter-extension in fractures of the inferior extremity. This is now accomplished successfully in three principle modes:—

1st. By the single inclined plane. A simple board or fracture-box being placed under the fractured limb, and sloping upward from the nates to the foot-board of the bed, is the principal apparatus. Adhesive straps are attached to the leg and fasten to a cord which runs over a pulley in the end of the board with a weight attached; this makes the extension. The weight of the body is the counter-extension. The same counter-extension can be made on the bed alone, by simply raising the foot of it ten or twelve inches, and placing the pully in the foot-board.

Probably for general use this is the best of all methods. Two other plans, however, may be resorted to. One is to have a long splint and a steel bow at the top reaching above the shoulder, which is attached to large adhesive straps, running down the breast and back. The other way is to make use of pressure against the ischium of the sound hip, as a *point d'appui* for counter-extension, as in a very ingenious apparatus devised by Dr. LATTA, of Goshen, Indiana.

The treatment of indolent ulcerations has apparently received an impetus from a suggestion of Dr. LUCIUS CLARK, of Rockford. The plan consists of simply giving the patient large and frequent doses of sulphur, internally, until the system is saturated with it. As tested by Prof. ANDREWS, at Mercy Hospital, in Chicago, the effect of the drug would seem to be to stimulate very powerfully the growth of granulations. One case took 30 grains at a dose, five times a day. In five days a luxuriant crop of granulations was produced in an indolent old ulcer of two years' standing. In ten days the ulcer was full and partly healed. In a few days more the cicatrization was complete. The granulations had a much redder color than usual, but did not present any other evidence of inflammation. If sulphur shall prove to have a reliable power of increasing the growth of granulations, it will be a most valuable agent. At present only about ten cases have been experimented upon. The success is such as to encourage further trial, but from so limited an experience, we do not feel willing to pronounce a final opinion upon the subject. It should be remarked that many cases require opiates with the sulphur, in order to restrain the purgative action.

The subject of the exsection of portions of nerves, for old and otherwise incurable neuralgias of aggravated character, has received some recent attention in this State. Some cases of ten years standing have been treated in Mercy Hospital with success. In one case the myloid branch of the inferior dental nerve, which has been commonly supposed to be a motor nerve, was the seat of trouble, and the removal of half an inch of it effected a cure, showing that it has, at least in part, a sensory function. In all such operations it is important to remember that the cause of the neuralgia is situated, not where the pain is felt, but in some foramen, canal, or notch through which the nerve passes, and in which the trunk of it is compressed by organic changes. The nerve, therefore, must be cut on the *proximal* side of such constrictions. For instance, the inferior dental must be cut by trephining the ramus of the jaw, and reaching it before it enters the bone. The direction of GROSS

to cut such nerves just at their exit from their foramina, is not only absurd, but is proved by experience to be utterly inefficient. It is only in a few terrible, and unusual cases of neuralgia, that the resections should be practiced.

E. ANDREWS, M.D.,
S. W. NOBLE, M.D.,
F. B. HALLER, M.D.,
*Committee on Surgery.*

